# Exploring Pharmacological Treatments for Chronic Idiopathic Constipation in Adults: A Look Back to the Future

**DOI:** 10.3390/jcm12041702

**Published:** 2023-02-20

**Authors:** Gabrio Bassotti, Vincenzo Villanacci, Maura Corsetti

**Affiliations:** 1 Gastroenterology, Hepatology & Digestive Endoscopy Section, Department of Medicine and Surgery, University of Perugia, 06123 Perugia, Italy; 2 Pathology Institute, ASST-Spedali Civili, 25123 Brescia, Italy; 3 National Institute for Health Research, Nottingham Biomedical Research Centre, Nottingham University Hospitals NHS Trust UK, School of Medicine, University of Nottingham and Nottingham Digestive Diseases Centre, University of Nottingham, Nottingham NG7 2RD, UK

**Keywords:** chronic constipation, alternative treatment, drug treatment, pharmacologic treatment, laxatives, magnesium salts

## Abstract

Despite great progress in pharmaceutical research, the medical treatment of chronic idiopathic constipation is far from ideal. The aim of the present article was to review literature data, focusing on poorly studied or commercially unavailable/unapproved drugs potentially useful for the treatment of chronic idiopathic constipation in adults. An extensive online literature search was conducted using the keywords “chronic constipation”, “colon”, “constipation”, “drugs”, “laxatives”, and “treatment”, in various combinations between January 1960 and December 2022. The literature search showed the presence of some drugs whose efficacy has only recently been demonstrated by modern investigations, and which are likely to be incorporated into future guidelines, of others that are proven effective and potentially effective on constipated patients but limited by small or relatively old studies, or by side effects which could be used in experienced hands, and of others that might be useful but lack a solid scientific background. Looking into the future for patients with chronic constipation might add some more tools to the therapeutic portfolio, especially for certain subgroups of these patients.

## 1. Introduction

Ask, and it shall be given you;

seek, and ye shall find;

knock, and it shall been opened

unto you

(Matthew, 7:7)

Chronic idiopathic constipation (CIC) is a frequently encountered disorder in daily clinical practice, and it is responsible for a considerable loss of productivity and health care utilization [1]. The medical treatment of CIC is usually based on the use of various types of laxatives [2,3] and several pharmacological approaches are also available for patients refractory to common first-line therapies [4]. Laxatives have a long history of use for the treatment of CIC [5]; interestingly, a meta-analysis in 2002 concluded that there was no objective evidence of their effective use for this indication, due to the low-quality studies concerning this topic [6]. However, in the following years, research interest has flared up in this area; thus, several double-blind randomized controlled trials have appeared in the literature, and there are now several drugs with laxative properties that are considered as an effective means of treating CIC [7].

However, recent evidence suggests that, when evaluated by rigorous means, there is a rather high percentage of patient dissatisfaction with the first- and second-line laxative-based treatment approaches [8], as suggested by Western and Eastern guidelines for CIC [9,10,11].

Although several new drugs active in CIC patients have been approved in recent years, it is worth emphasizing that a thorough analysis of the results from well-conducted double-blind randomized studies has shown that these drugs do not appear to be superior to older drugs [12], which are also considerably cheaper.

The aim of the present review was to evaluate the available literature focusing on poorly studied—or effective but not commercially available/not approved—drugs potentially useful for the treatment of patients with CIC.

## 2. Methods

Search strategy. An extensive search of Medline (through PubMed), Scopus, Cochrane CENTRAL, EMBASE, and the Science Citation Index was made using the keywords “chronic constipation”, “colon”, “constipation”, “drugs”, “laxatives”, and “treatment”, in various combinations with the Boolean operators and, or, and not. Only articles from adult human studies were included and manual cross-referencing was performed. Articles published in English between January 1960 and January 2023 were selected, but a search was also conducted in non-English languages and among journals and books older than 1960 in our University and other libraries.

## 3. Commercially Available Drugs with Evidence of Efficacy on CIC

### 3.1. Magnesium Salts

Pharmaceutical preparations containing magnesium (magnesium nitrate, magnesium sulfate, magnesium oxide, magnesium hydroxide, and magnesium citrate) have been in use in Eastern and Western countries since at least the 8th century [13]. However, the use of magnesium salts was more based on anecdotal than on actual scientific evidence [14]. Some small pediatric studies showed that magnesium hydroxide was as effective as macrogol in relieving symptoms in children [15,16], but no data were available on adult CIC patients. More recently, due to its safety, availability and substantially low cost (at least compared to other laxatives), there has been renewed interest in magnesium salts. Two randomized clinical trials on the effects of magnesium oxide in adult CIC patients have recently appeared in the literature. In a double-blind randomized controlled trial patients received magnesium oxide (0.5 g t.i.d.) or a placebo for 28 days; the first group had a significant improvement in overall constipation scores compared to the placebo (70.6% vs. 25%, *p* = 0.015), a significant overall increase in spontaneous bowel movements (SBM, 6.07 ± 2.26 vs. 2.86 ± 2.42, *p* = 0.002) and an improvement in the quality of life, as well as in colonic transit time [17]. In a second randomized, placebo-controlled, double-blind trial, patients received magnesium oxide (1.5 g per os), senna (1 g) or a placebo for 28 days. Analysis of the study data revealed that an overall improvement in symptoms was documented in 68.3% of patients who received magnesium oxide, 69.2% of those who received senna, and 11.7% of those who received the placebo (*p* < 0.0001) [18]. Compared to the placebo, SBM increased significantly in patients taking magnesium and senna (*p* < 0.001), and the same was documented for complete SBM (*p* < 0.01). Furthermore, significant improvements in the quality of life were recorded for senna (*p* < 0.05) and magnesium (*p* < 0.001) compared to the placebo. No significant adverse events were reported for both laxatives.

Interestingly, despite the long history of treating CIC with natural mineral waters rich in magnesium sulfate [19], no controlled studies on this topic were available until recently. Some recent studies, however, have demonstrated the benefits of this treatment in constipated subjects. In a randomized, double-blind, placebo-controlled trial 244 constipated women (Rome III criteria) were randomized to drink 1.5 L of natural low-mineral water (controls), 0.5 L of magnesium sulfate-rich natural mineral water (Hépar) plus 1 L of natural low-mineral water, or 1 L of Hépar plus 0.5 L of natural low-mineral water daily for a period of four weeks [20]. Information was obtained on the number and type of stools (according to the Bristol scale), abdominal pain, adverse events, and rescue medications. Analysis of the results showed that after the first week no changes in bowel parameters occurred. After two weeks, constipation improved in about 20% of the controls, in about 31% of the Hépar 0.5 L group, and in about 38% in the Hépar 1 L group; both Hépar groups showed significant differences compared to the controls. The Hépar 1 L group also showed a significant decrease in fecal consistency and the use of rescue drugs compared to the controls. The safety of this approach was rated as very good by the investigators, and no serious adverse events were reported [20]. These results were confirmed by a subsequent investigation carried out by the same group in 226 patients, randomized to drink 1.5 L of natural low-mineral water (controls), or 1 L Hépar plus 0.5 L of natural low-mineral water over a two-week period [21]. Again, no safety concerns were raised and the response time for symptom improvement was one week. Another randomized, double-blind, placebo-controlled study of 100 patients with CIC (Rome III criteria) evaluated the effects of magnesium sulfate-rich mineral water (Ensinger Schiller Quelle), 1 L per day, compared to the same amount of carbonated tap water (as placebo) over a period of six weeks [22]. The primary endpoint was the change in frequency of evacuations/week between baseline and the end of the study, while the secondary endpoint was the change in frequency of evacuations/week between baseline and three weeks. Analysis of the results showed that after six weeks of treatment no significant differences were appreciated between the two groups; however, at three weeks significant differences were found in the active treatment group compared to placebo (2.02 ± 2.22 vs. 0.88 ± 1.67 defecations/week, *p* = 0.005), suggesting that the effect of this mineral water was time-limited. Another randomized, double-blind, placebo-controlled study examined the effects of another mineral water rich in magnesium sulfate (Donat) on 106 CIC patients (Rome III criteria) over a period of six week [23]. Patients were randomized into four arms, two drinking 300 mL/day of Donat or low-mineral sparkling water (placebo), and two drinking either 500 mL/day of Donat or a placebo as above. Data analysis showed no significant benefit of Donat in the 300 mL arm, which was closed after the interim analysis, whereas in the 500 mL arm, patients drinking Donat displayed significant improvements at the end of the study period compared to the placebo in the number of complete spontaneous bowel movements (*p* = 0.036), stool consistency (*p* < 0.01), and subjectively perceived constipation symptoms (*p* = 0.005). The treatment was considered safe overall by the investigators.

To date, the use of magnesium oxide in the treatment of patients with CIC is only mentioned in Japanese guidelines, with a “strong” recommendation [13,24]. Other magnesium salts are mentioned in other guidelines, but with a “weak” recommendation due to the low level of evidence for these combinations [13].

### 3.2. Colchicine

Colchicine is a natural alkaloid with a long historical use in medicine for the treatment of inflammatory diseases, such as gout; its effects on gastrointestinal transit acceleration have been documented for centuries [25]. A recent systematic review confirmed that colchicine increases the rate of gastrointestinal adverse events, especially diarrhea [26]. The latter fact, well-known among physicians, stimulated the interest of researchers as a potentially useful effect in patients with CIC. Thus, after a preliminary report on its efficacy in three severely constipated parkinsonian patients [27], colchicine was tested in a small uncontrolled/pilot study to treat CIC patients refractory to conventional medical therapy. Verne and colleagues treated seven of these patients with colchicine, 0.6 mg per os t.i.d. for two months, documenting a significant increase in SBM compared to baseline (6.4 ± 0.7 vs. 1.7 ± 0.5, *p* < 0.05) [28]. Subsequently, two randomized, placebo-controlled trials were conducted on severely constipated patients who did not respond to medical treatment. In the first study 16 patients were randomized to receive 0.6 mg colchicine t.i.d. or a placebo for a month [29]. Compared to the placebo and baseline, colchicine significantly increased the number of weekly bowel movements (2.7 ± 1.8 vs. 9.9 ± 5.3, *p* < 0.0001) and accelerated colonic transit (63.1 ± 12.9 vs. 29.1 ± 19.1 h, *p* < 0.0001). In the second study 60 patients (30 in each group) were randomized to receive colchicine (1 mg q.i.d.) or a placebo for two months [30]. At the end of the study period, the symptom score (Knowles–Eccersley–Scot score) was significantly reduced for colchicine compared to the placebo (11.7 ± 4 vs. 18.7 ± 4, *p* = 0.0001).

### 3.3. Misoprostol

An analogue of prostaglandin E1, misoprostol is sometimes used in gastroenterology as a preventive agent against the adverse effects of non-steroidal anti-inflammatory drugs [31]. Due to its effects on accelerating gastrointestinal transit [32,33], misoprostol often causes diarrhea, especially at higher doses, and this effect has been exploited as a possible treatment for CIC [34]. Two small studies were carried out in patients with severe symptoms and refractory to other treatments. The first was a three-week double-blind, randomized, crossover study of nine patients [35]. Compared to the placebo, misoprostol (1200 mcg/day) significantly increased the number of weekly evacuations (6.5 ± 1.3 vs. 2.5 ± 0.11, *p* = 0.001), total weekly stool weight (976.5 ± 289 g vs. 434.6 ± 190.5 g, *p* = 0.001), and large bowel transit time (66 ± 10.2 h vs. 109.4 ± 8 h, *p* = 0.0005). The second open-label study lasted four weeks and was conducted in 18 CIC patients with refractory symptoms, who were administered misoprostol (600–2400 mcg/day) as an adjunctive therapy [36]. As six patients dropped out of the study due to adverse events (cramps and abdominal discomfort), data were obtained from the remaining 12; in these patients a significant reduction in the mean interval between the frequency of defecations compared to baseline was reported (4.8 vs. 11.2 days, *p* = 0.0004). In a small subgroup of patients (N = 4) in this study, the effect of a single dose (400 mcg/day) of the drug on post-prandial colonic motor activity was evaluated and compared with results obtained in five healthy controls. Misoprostol significantly increased the colonic motor response to the meal in the whole colon, with a greater response in the left than in the right segments of the large bowel. Despite the potential usefulness of misoprostol for the treatment of constipated subjects, due to its abortive effects [37] and the fact that most patients were women, this drug has not been further exploited in other randomized controlled trials for the treatment of CIC.

### 3.4. Antibiotics

Although there is recent evidence that the gut microbiome may be abnormal and play an important clinical role in CIC patients [38,39,40], the effects on its imbalance caused by various factors (including antibiotic therapies [41]) have only been explored in a limited number of studies. In a small uncontrolled study, eight women with CIC resistant to dietary fibers were given ispaghula for a fortnight, followed by oral vancomycin (250 mg t.i.d.) for two further weeks while continuing to receive the fiber supplement [42]. Daily bowel symptoms (diary) and stools were collected during the two study periods. Whole gut transit time and the oro–caecal transit time (breath hydrogen test) after a standard meal were measured at the end of each period together with gastrointestinal symptoms (visual analogue scale). Administration of vancomycin significantly increased the frequency and improved the consistency of stools, ease of defecation and the amount of stool the patients felt they were producing. However, objective measures of stool weight and whole or oro–caecal intestinal transit time showed no significant differences.

In a controlled pilot study, 30 patients with CIC unresponsive to dietary fibers were randomized to receive oral lincomycin (500 mg) and fibers or a placebo plus fibers for 10 days, followed by a 10-day period in which they only received fibers [43]. In the lincomycin group, the frequency of weekly defecations increased from 2.6 to 4.4 (*p* < 0.02), while it remained unchanged (2.9) in the placebo group.

Another pilot study, randomized and placebo-controlled, investigated the effect of rifaximin (400 mg t.i.d.) on the colonic transit and methane production of 23 CIC patients [44]. After 14 days of treatment, rifaximin-treated patients had a significantly reduced colonic transit compared to the placebo, while the weekly stool frequency (diary) and form (Bristol stool scale) tended to improve, and methane production was reduced.

Two studies conducted by the same group showed that eradication of *Helicobacter pylori* in patients with associated CIC can improve constipation symptoms. In a short-term study, 166 patients underwent eradication (vonoprazan plus amoxicillin/clarithromycin or amoxicillin/metronidazole, or amoxicillin/sitafloxacin) and constipation-related symptoms were assessed with the gastrointestinal symptom rating scale score [45]. In patients with successful eradication, scores were significantly improved two months after eradication, compared to baseline (8.00 ± 2.8 vs. 6.16 ± 3, *p* < 0.01), while scores in patients with failed eradication were similar before and after eradication. The same group carried out another such study over a long-term period (2 and 12 months) [46]. Two hundred and seventy eight *Helicobacter pylori*-positive patients underwent eradication as in the first study. Constipation-related scores, measured as above, showed that successfully eradicated patients improved significantly compared to baseline two months (7.91 ± 3.15 vs. 6.07 ± 2.75, *p* < 0.01) and one year after treatment (6.85 ± 3.46, *p* = 0.04). In patients with improved scores two months after treatment, an improvement one year after treatment was observed. In contrast, patients without an improvement after two months did not show an improvement after one year.

### 3.5. Pyridostigmine

Acetylcholinesterase inhibitors, such as neostigmine and pyridostigmine, delay the degradation of acetylcholine at the synaptic cleft. This increase in acetylcholine has been shown to increase gut motility, which has led to their use in dysmotility of the gastrointestinal tract [47], including CIC.

In a uncontrolled pilot study, 10 CIC patients with autonomic neuropathy were treated with a placebo for a fortnight and then given pyridostigmine up to the maximum tolerated dose (180 to 540 mg/day) for six weeks [48]. Analysis of the results showed that the drug was well-tolerated in most patients, but symptoms (severity scores of constipation) improved in only 40% of patients, and colonic transit was accelerated in only 30% of cases. In another small uncontrolled study, six patients with CIC were initially given 10 mg b.i.d. of pyridostigmine, increased to 30 mg b.i.d. for several weeks if the initial dose was ineffective [49]. Only one constipated patient showed transient benefits from the treatment. In a further randomized controlled investigation, 30 CIC constipated patients with diabetes mellitus (18 type 1, 12 type 2) were given either a placebo or pyridostigmine (60 mg t.i.d. at baseline and increased by 60 mg every third day up to the maximum tolerated dose or 120 mg t.i.d., maintaining this dose for a week) [50]. Patients were evaluated clinically and by gastrointestinal and colonic transit scintigraphy at baseline and on the last three and seven days of treatment. Analysis of the results showed that pyridostigmine significantly improved daily stool frequency (0.95 ± 0.2 vs. 1.5 ± 0.2, *p* = 0.02), consistency (Bristol scale, 2.5 ± 0.3 vs. 3.4 ± 0.2, *p* < 0.005), and ease of stool transit (3.5 ± 0.2 vs. 3.8 ± 0.5, *p* < 0.04). In addition, the drug significantly accelerated colonic transit after 24 h (1.96 ± 0.18 vs. 2.45 ± 0.20, *p* < 0.01), but showed no significant effects compared to the placebo with regard to gastric or small bowel transit.

A more recent double-blind study compared the effects of pyridostigmine and bisacodyl in CIC patients refractory to conventional treatments. For this purpose, 68 of these patients (34 per group) were randomly assigned to pyridostigmine (60 mg t.i.d.) or bisacodyl (5 mg t.i.d.) for four weeks [51]. Compared to baseline, the number of weekly defecations improved significantly in both the pyridostigmine group (1.55 ± 1.28 vs. 5.96 ± 1.84, *p* = 0.005) and the bisacodyl group (2.26 ± 1.48 vs. 5.16 ± 1.95, *p* = 0.005).

### 3.6. Trimebutine

Trimebutine maleate is a spasmolytic drug that acts on the gastrointestinal tract through an agonist effect on peripheral mu, kappa and delta opioid receptors, the release of gastrointestinal peptides (motilin), and modulation of the release of other peptides (gastrin, glucagon, and vasoactive intestinal peptide) [52]. The drug has been shown to be effective in stimulating colonic motility in experimental animals [53].

The effects of trimebutine in patients with CIC were analyzed in a double-blind crossover study of 24 patients. Fecal frequency, colonic transit time, and electromyographic activity of the large intestine were evaluated at baseline and after receiving trimebutine (200 mg per day) or a placebo for one month [54]. Compared to the placebo, (a) stool frequency did not differ after trimebutine treatment, although both significantly increased stool frequency, suggesting a placebo effect on this variable; (b) colonic transit time decreased significantly (from 105 ± 19 to 60 ± 11 h) only in patients with delayed transit; (c) trimebutine, again in patients with delayed transit, significantly increased the number of post-prandial propagating burst (from 2.1 +/− 0.3 bursts/h to 3.5 +/− 0.6 bursts/h), events associated with the colonic transport of contents and defecatory stimuli [55].

## 4. Other Drugs with Possible Efficacy on CIC

### 4.1. Neurotrophin-3

Neurotrophins are growth factors that regulate the development and repair of the nervous system, that are involved in the pathogenesis of some neurodegenerative diseases, and have promising therapeutic potential on nervous system diseases related to vascular lesions and pathology, such as neuropsychiatry, neurodegeneration and peripheral nerve diseases [56]. After neuroptrophin-3 (NT-3) was shown to stimulate gastrointestinal (in particular colonic) motility in experimental animal models [57], it was tested on the symptoms, colonic transit, and intestinal function of CIC patients. In a phase II, randomized, controlled double-blind study 107 patients with CIC (Rome II criteria) were randomized to receive double-blind subcutaneous injections containing a placebo, 3 mg, then 9 mg NT-3 once or three times a week, or 9 mg NT-3 three times a week, then once a week, over a period of four weeks [58]. Patients treated with 9 mg NT-3 three times a week showed a significant increase in total bowel movements and in the frequency of spontaneous and complete bowel movements, as well as a dose-related softening of stools and improved ease of passage compared to the placebo. In these patients, the number of days per week without a bowel movement also decreased, and constipation-related symptoms and colon transit improved. In contrast, weekly administration was ineffective. The most frequent adverse events were represented by transient injection-site reactions, observed in one third of patients receiving NT-3.

### 4.2. Orlistat

The anti-obesity drug orlistat is a potent and specific intestinal lipase inhibitor used in weight reduction programs due to its efficacy [59]. The use of orlistat has been associated with diarrhea [60]. It is therefore not surprising that researchers have exploited this drug to treat CIC patients. An early report described three cases of severe, intractable CIC that benefitted, at least in the short-term period, from the administration of orlistat after other treatment regimens had failed [61]. In another study, seven overweight women with CIC, who did not respond to laxatives and were ineligible for surgery, were treated with orlistat, 120 mg t.d.s. Five of them continued the drug, and four of them (80%) reported significant improvement in constipation symptoms [62].

Table 1 summarizes the review results on the possible usefulness of alternative pharmacological treatments for patients complaining of CIC. It is worth remembering that only magnesium salts and magnesium sulfate-rich mineral waters are commercially available as over-the-counter formulations to treat constipation, and that, to date, no other approved drugs exist concerning CIC treatment.

## 5. Conclusions

In conclusion, a thorough literature search has shown that there might be a rethink on the use of a few old, relatively inexpensive and effective drugs for CIC patients, such as some magnesium salts, while many others need more accurate and modern investigations before they can be proposed for this purpose. There is little doubt that the pharmacologic approach to the treatment of CIC, despite important advances in drug development [11,12], is still far from an ideal goal [8,63]. Furthermore, patients’ knowledge on this issue is hampered by the poor quality and readability of online information [64]. New drugs, besides not being the much hoped for “magic bullets”, are considerably expensive, limiting the possibility for patients to be treated [65]. It is therefore perhaps time to look back into the future, to see if there is room for further possibilities due to old or poorly studied drugs, or mild-to-moderate adverse events of drugs developed for other purposes [66]. This literature review underlined how there may be room and scope for some of the abovementioned agents, which have been repeatedly studied and proven to be potentially useful. For example, magnesium salts (which are relatively inexpensive) have recently been investigated with up-to-date methods and have been shown to be effective in treating CIC patients. Therefore, we believe that these agents, particularly magnesium oxide and magnesium sulfate, should be added to the routine therapeutic armamentarium for the initial treatment of CIC patients. Other agents might have a new life under particular circumstances: colchicine and pyridostigmine, in expert hands, might represent additional effective weapons for subgroups of constipated subjects (e.g., those with delayed transit and refractory symptoms, and scleroderma patients [67]). The main problem with these drugs is the potential side effects and the fact that the scientific evidence supporting their use is relatively weak, as the relevant studies have been conducted on small groups of patients for short periods of time. Since other adverse events are comparatively infrequent [26], colchicine might be considered as an alternative option in selected constipated patients with constipation refractory to conventional treatments, at least in the short-term and in this clinical setting, although its long-term use (as seen in other pathological conditions) appears to be relatively safe [68]. Although pyridostigmine might be useful in the treatment of patients with CIC, this treatment carries the risk of major adverse events [69] and limits its use in clinical practice until more evidence is available from larger, well-conducted studies.

As for misoprostol, again taking into account the above warnings, its use could be restricted to male patients with delayed transit refractory to other treatments.

On the other hand, although there are numerous other drugs in the literature that have been suggested as effective for the treatment of CIC, the lack of objective scientific evidence, due to the heterogeneity of the treatments, the small groups of patients studied, the different entry criteria for these studies, and the limited period of investigation, prevents its use as an occasional treatment. Interestingly, however, antibiotic treatment, with its influence on the gut microbiota, appears to be particularly attractive and likely to be exploited by researchers in the near future.

In conclusion, a thorough literature search evidenced that there might be some rethinking on the use of a few old, relatively inexpensive and effective drugs for CIC patients, such as some magnesium salts, whereas several others will need more accurate and modern investigations before being proposed for this purpose.

## Figures and Tables

**Table 1:** Potential alternative treatments for chronically constipated patients.

Drug	Effectiveness on CIC (Scientific Evidence—Expert Opinion) [Refs]	Clinical Trials [Refs]		Possible Use
Magnesium oxide	Strong [17,18,20,21,22,23]	RCT [17] [18] [20] [21] [22] [23]	0.5 g t.i.d. (28 days) 1.5 g/day (28 days) 0.5/1 L/day (4 weeks) 1 L/day (2 weeks) 1 L/day (6 weeks) 300/500 mL/day (6 weeks)	All patients
Colchicine	Weak [27,28,29,30]	Case report [27] Uncontrolled pilot study [28] RCT: [29] [30]	0.3–0.6 mg/day 0.6 mg t.i.d. (2 months) 0.6 mg t.i.d. (1 months) 1 mg q.i.d. (1 months)	STC patients, refractory to treatment
Misoprostol	Weak [35,36]	RCT [35] Open label study [36]	1200 mcg/day (3 weeks) 600–2400 mcg/day (4 weeks)	STC male patients, refractory to treatment
Antibiotics	Weak [42,43,44,45,46]	Small uncontrolled study [42] RCT [43] [44] Observational study [45,46]	Vancomycin 250 mg t.i.d. (2 weeks) Lincomycin 500 mg/day (10 days) Rifaximin 400 mg t.i.d. (2 weeks) Amoxicillin (750 mg bid) and clarithromycin (200 mg b.i.d.) as first-line treatment, amoxicillin (750 mg, b.i.d.) and metronidazole (250 mg, b.i.d.) as second-line treatment, and amoxicillin (500 mg, q.i.d.) and sitafloxacin (100 mg, b.i.d.) as third- or fourth-line treatment, all for 7 days	All patients
Pyridostigmine	Weak [48,49,50,51]	Uncontrolled study [48] [49] RCT [50] [51]	180–540 mg/day (6 weeks) 10 mg/day b.i.d. (4 weeks) 60–120 mg/day (1 weeks) 60 mg t.i.d. (4 weeks)	Selected subgroups (e.g., scleroderma patients)
Trimebutine	Weak [54]	RCT [54]	200 mg/day (1 month)	Patients with delayed transit
Neurotrophin-3	Weak [58]	RCT [58]	3–9 mg/week, or 9 mg 1–3 times/week	All patients
Orlistat	Weak [61,62]	Case report [61] Small uncontrolled study [62]	60–120 mg t.id. (2 weeks) 120 mg t.i.d. (6 weeks)	Obese patients

Apart from magnesium sulfate, available in many Western and Eastern countries, and magnesium-rich mineral waters, available in some European Countries (France, Germany, Slovakia), the other drugs are not yet approved for the treatment of CIC in any country. Abbreviations: CIC = chronic idiopathic constipation; RCT = randomized clinical trial; STC = slow transit constipation.

## Data Availability

No new data were created for the review.

## References

[1] Neri L., Basilisco G., Corazziari E., Stanghellini V., Bassotti G., Bellini M., Perelli I., Cuomo R., LIRS Study Group (2014). Constipation severity is associated with productivity losses and healthcare utilization in patients with chronic constipation. United Eur. Gastroenterol. J..

[2] Bassotti G., Villanacci V. (2013). A practical approach to diagnosis and management of functional constipation in adults. Intern. Emerg. Med..

[3] Bellini M., Usai-Satta P., Bove A., Bocchini R., Galeazzi F., Battaglia E., Alduini P., Buscarini E., Bassotti G., ChroCoDiTE Study Group AIGO (2017). Chronic constipation diagnosis and treatment evaluation: The “CHRO.CO.DI.T.E.” study. BMC Gastroenterol.

[4] Bassotti G., Blandizzi C. (2014). Understanding and treating refractory constipation. World J. Gastrointest. Pharmacol. Ther..

[5] Capasso L. (1998). 5300 years ago, the Ice Man used natural laxatives and antibiotics. Lancet.

[6] Jones M.P., Talley N.J., Nuyts G., Dubois M. (2002). Lack of objective evidence of efficacy of laxatives in chronic constipation. Dig. Dis. Sci..

[7] Pannemans J., Masuy I., Tack J. (2020). Functional Constipation: Individualising Assessment and Treatment. Drugs.

[8] Basilisco G., Italian Society of Neurogastroenterology Motility (SINGEM) Study Group (2020). Patient dissatisfaction with medical therapy for chronic constipation or irritable bowel syndrome with constipation: Analysis of N-of-1 prospective trials in 81 patients. Aliment. Pharm..

[9] Shin J.E., Jung H.K., Lee T.H., Jo Y., Lee H., Song K.H., Hong S.N., Lim H.C., Lee S.J., Chung S.S. (2016). Guidelines for the diagnosis and treatment of chronic functional constipation in Korea, 2015 revised edition. J. Neurogastroenterol Motil..

[10] Serra J., Pohl D., Azpiroz F., Chiarioni G., Ducrotté P., Gourcerol G., Hungin A.P.S., Layer P., Mendive J.M., Pfeifer J. (2020). European society of neurogastroenterology and motility guidelines on functional constipation in adults. Neurogastroenterol Motil.

[11] Bassotti G., Satta P.U., Bellini M. (2021). Chronic Idiopathic Constipation in Adults: A Review on Current Guidelines and Emerging Treatment Options. Clin. Exp. Gastroenterol..

[12] Luthra P., Camilleri M., Burr N.E., Quigley E.M.M., Black C.J., Ford A.C. (2019). Efficacy of drugs in chronic idiopathic constipation: A systematic review and network meta-analysis. Lancet Gastroenterol. Hepatol..

[13] Mori H., Tack J., Suzuki H. (2021). Magnesium Oxide in Constipation. Nutrients.

[14] Tack J., Müller-Lissner S., Stanghellini V., Boeckxstaens G., Kamm M.A., Simren M., Galmiche J.-P., Fried M. (2011). Diagnosis and treatment of chronic constipation-a European perspective. Neurogastroenterol. Motil..

[15] Loening-Baucke V., Pashankar D.S. (2006). A Randomized, Prospective, Comparison Study of Polyethylene Glycol 3350 without Electrolytes and Milk of Magnesia for Children with Constipation and Fecal Incontinence. Pediatrics.

[16] Gomes P.B., Duarte M.A., Melo Mdo C. (2010). Comparison of the effectiveness of polyethylene glycol 4000 without electrolytes and magnesium hydroxide in the treatment of chronic functional constipation in children. J. Pediatr..

[17] Mori S., Tomita T., Fujimura K., Asano H., Ogawa T., Yamasaki T., Kondo T., Kono T., Tozawa K., Oshima T. (2019). A Randomized Double-blind Placebo-controlled Trial on the Effect of Magnesium Oxide in Patients with Chronic Constipation. J. Neurogastroenterol. Motil..

[18] Morishita D., Tomita T., Mori S., Kimura T., Oshima T., Fukui H., Miwa H. (2020). Senna Versus Magnesium Oxide for the Treatment of Chronic Constipation: A Randomized, Placebo-Controlled Trial. Am. J. Gastroenterol..

[19] Dupont C., Hébert G. (2020). Magnesium Sulfate-Rich Natural Mineral Waters in the Treatment of Functional Constipation–A Review. Nutrients.

[20] Dupont C., Campagne A., Constant F. (2014). Efficacy and Safety of a Magnesium Sulfate–Rich Natural Mineral Water for Patients with Functional Constipation. Clin. Gastroenterol. Hepatol..

[21] Dupont C., Constant F., Imbert A., Hébert G., Zourabichvili O., Kapel N. (2019). Time to treatment response of a magnesium- and sulphate-rich natural mineral water in functional constipation. Nutrition.

[22] Naumann J., Sadaghiani C., Alt F., Huber R. (2016). Effects of Sulfate-Rich Mineral Water on Functional Constipation: A Double-Blind, Randomized, Placebo-Controlled Study. Complement. Med. Res..

[23] Bothe G., Coh A., Auinger A. (2015). Efficacy and safety of a natural mineral water rich in magnesium and sulphate for bowel function: A double-blind, randomized, placebo-controlled study. Eur. J. Nutr..

[24] Research Society for the Diagnosis and Treatment of Chronic Constipation Affiliated to the Japanese Society of Gastroenterology (2017). Evidence-Based Clinical Practice Guideline for Chronic Constipation.

[25] Ahern M.J., Reid C., Gordon T., McCredle M., Brooks P.M., Jones M. (1987). Does colchicine work? The results of the first controlled study in acute gout. Aust. N. Z. J. Med..

[26] Stewart S., Yang K.C.K., Atkins K., Dalbeth N., Robinson P.C. (2020). Adverse events during oral colchicine use: A systematic review and meta-analysis of randomised controlled trials. Arthritis. Res. Ther..

[27] Sandyk R., Gillman M.A. (1984). Colchicine ameliorates constipation in Parkinson’s disease. J. R. Soc. Med..

[28] Verne G.N., Eaker E.Y., Davis R.H., Sninsky C.A. (1997). Colchicine is an effective treatment for patients with chronic constipation: An open-label trial. Dig. Dis. Sci..

[29] Verne N.G., Davis R.H., Robinson M.E., Gordon J.M., Eaker E.Y., Sninksy C.A. (2003). Treatment of Chronic Constipation with Colchicine: Randomized, Double-Blind, Placebo-Controlled, Crossover Trial. Am. J. Gastroenterol..

[30] Taghavi S.A., Shabani S., Mehramiri A., Eshraghian A., Kazemi S.M.H., Moeini M., Hosseini-Asl S.M.K., Saberifiroozi M., Alizade-Naeeni M., Mostaghni A.A. (2010). Colchicine is effective for short-term treatment of slow transit constipation: A double-blind placebo-controlled clinical trial. Int. J. Color. Dis..

[31] Guo C.-G., Leung W.K. (2020). Potential Strategies in the Prevention of Nonsteroidal Anti-inflammatory Drugs-Associated Adverse Effects in the Lower Gastrointestinal Tract. Gut Liver.

[32] Rutgeerts P., Vantrappen G., Hiele M., Ghoos Y., Onkelinx C. (1991). Effects on bowel motility of misoprostol administered before and after meals. Aliment. Pharmacol. Ther..

[33] Soffer E.E., Launspach J. (1993). Effect of misoprostol on postprandial intestinal motility and orocecal transit time in humans. Dig. Dis. Sci..

[34] Davies N.M., Longstreth J., Jamali F. (2001). Misoprostol Therapeutics Revisited. Pharmacotherapy.

[35] Soffer E.E., Metcalf A., Launspach J. (1994). Misoprostol is effective treatment for patients with severe chronic constipation. Dig. Dis. Sci..

[36] Roarty T.P., Weber F., Soykan I., McCallum R.W. (1997). Misoprostol in the treatment of chronic refractory constipation: Results of a long-term open label trial. Aliment. Pharmacol. Ther..

[37] Zhang J., Zhou K., Shan D., Luo X. (2022). Medical methods for first trimester abortion. Cochrane Database Syst. Rev..

[38] Cao H., Liu X., An Y., Zhou G., Liu Y., Xu M., Dong W., Wang S., Yan F., Jiang K. (2017). Dysbiosis contributes to chronic constipation development via regulation of serotonin transporter in the intestine. Sci. Rep..

[39] Yarullina D.R., Shafigullin M.U., Sakulin K.A., Arzamastseva A.A., Shaidullov I.F., Markelova M.I., Grigoryeva T.V., Karpukhin O.Y., Sitdikova G.F. (2020). Characterization of gut contractility and microbiota in patients with severe chronic constipation. PLoS ONE.

[40] Tian H., Chen Q., Yang B., Qin H., Li N. (2020). Analysis of Gut Microbiome and Metabolite Characteristics in Patients with Slow Transit Constipation. Dig. Dis. Sci..

[41] Yang L., Bajinka O., Jarju P.O., Tan Y., Taal A.M., Ozdemir G. (2021). The varying effects of antibiotics on gut microbiota. AMB Express.

[42] Celik A.F., Tomlin J., Read N.W. (1995). The effect of oral vancomycin on chronic idiopathic constipation. Aliment. Pharmacol. Ther..

[43] Fernández L.M.B., Prizont R., Soifer L.O. (2013). A controlled pilot study on the efficacy of a low dose antibiotic for the treatment of chronic constipation in patients receiving a high fiber diet. Acta Gastroenterol. Latinoam..

[44] Ghoshal U.C., Srivastava D., Misra A. (2018). A randomized double-blind placebo-controlled trial showing rifaximin to improve constipation by reducing methane production and accelerating colon transit: A pilot study. Indian J. Gastroenterol..

[45] Murata M., Sugimoto M., Otsuka T., Nishida A., Inatomi O., Bamba S., Andoh A. (2018). Successful Helicobacter pylori eradication therapy improves symptoms of chronic constipation. Helicobacter.

[46] Murata M., Sugimoto M., Miyamoto S., Kawai T. (2022). Long-term improvement in constipation-related symptoms after Helicobacter pylori eradication therapy. Helicobacter.

[47] Traeger L., Kroon H.M., Bedrikovetski S., Moore J.W., Sammour T. (2021). The impact of acetylcholinesterase inhibitors on ileus and gut motility following abdominal surgery: A clinical review. ANZ J. Surg..

[48] Bharucha A.E., Low P.A., Camilleri M., Burton D., Gehrking T.L., Zinsmeister A.R. (2008). Pilot study of pyridostigmine in constipated patients with autonomic neuropathy. Clin. Auton. Res..

[49] O’Dea C.J., Brookes S., Wattchow D.A. (2009). The efficacy of treatment of patients with severe constipation or recurrent pseudo-obstruction with pyridostigmine. Color. Dis..

[50] Bharucha A.E., Low P., Camilleri M., Veil E., Burton D., Kudva Y., Shah P., Gehrking T., Zinsmeister A.R. (2013). A randomised controlled study of the effect of cholinesterase inhibition on colon function in patients with diabetes mellitus and constipation. Gut.

[51] Soufi-Afshar I., Moghadamnia A., Bijani A., Kazemi S., Shokri-Shirvani J. (2016). Comparison of pyridostigmine and bisacodyl in the treatment of refractory chronic constipation. Casp. J. Intern. Med..

[52] Delvaux M., Wingate D. (1997). Trimebutine: Mechanism of Action, Effects on Gastrointestinal Function and Clinical Results. J. Int. Med. Res..

[53] Buéno L., Hondé C., Pascaud X., Junien J.L. (1987). Effects of orally vs. parenterally administrated trimebutine on gastrointestinal and colonic motility in dogs. Gastroenterol. Clin. Biol..

[54] Schang J.C., Devroede G., Pilote M. (1993). Effects of trimebutine on colonic function in patients with chronic idiopathic constipation: Evidence for the need of a physiologic rather than clinical selection. Dis. Colon. Rectum..

[55] Corsetti M., Costa M., Bassotti G., Bharucha A.E., Borrelli O., Dinning P., Di Lorenzo C., Huizinga J.D., Jimenez M., Rao S. (2019). First translational consensus on terminology and definitions of colonic motility in animals and humans studied by manometric and other techniques. Nat. Rev. Gastroenterol. Hepatol..

[56] Omar N.A., Kumar J., Teoh S.L. (2022). Neurotrophin-3 and neurotrophin-4: The unsung heroes that lies behind the meninges. Neuropeptides.

[57] Chai N.-L., Dong L., Li Z.-F., Du K.-X., Wang J.-H., Yan L.-K., Dong X.-L. (2003). Effects of neurotrophins on gastrointestinal myoelectric activities of rats. World J. Gastroenterol..

[58] Parkman H.P., Rao S.S., Reynolds J.C., Schiller L.R., Wald A., Miner P.B., Lembo A.J., Gordon J.M., Drossman D.A., Waltzman L. (2003). Functional Constipation Study Investigators. Neurotrophin-3 improves functional constipation. Am. J. Gastroenterol.

[59] Khalil H., Ellwood L., Lord H., Fernandez R. (2020). Pharmacological Treatment for Obesity in Adults: An Umbrella Review. Ann. Pharmacother..

[60] Filippatos T.D., Derdemezis C.S., Gazi I.F., Nakou E.S., Mikhailidis D.P., Elisaf M.S. (2008). Orlistat-associated adverse effects and drug interactions: A critical review. Drug. Saf..

[61] Guarino A.H. (2005). Treatment of Intractable Constipation with Orlistat: A Report of Three Cases. Pain Med..

[62] Iqbal F., Samuel M., Tan E.J.K., Nicholls R.J., Vaizey C.J. (2016). Letter: Orlistat as a potential treatment for chronic idiopathic constipation. Aliment. Pharmacol. Ther..

[63] Nee J., Sugarman M.A., Ballou S., Katon J., Rangan V., Singh P., Zubiago J., Kaptchuk T.J., Lembo A. (2019). Placebo Response in Chronic Idiopathic Constipation: A Systematic Review and Meta-Analysis. Am. J. Gastroenterol..

[64] Gollapalli S., Bresler R., Lynch N.P., Martin S.T. (2022). Treatment for Constipation—An Online Search. Readability and Quality of Online Patient Resources. J. Patient Exp..

[65] Bassotti G. (2021). New pharmacologic treatments for idiopathic chronic constipation: A financial strain for strainers. Expert Rev. Gastroenterol. Hepatol..

[66] Domagała-Rodacka R., Cibor D., Szczeklik K., Rodacki T., Mach T., Owczarek D. (2016). Gastrointestinal tract as a side-effect target of medications. Przeglad Lek..

[67] Ahuja N.K., Mische L., Clarke J.O., Wigley F.M., McMahan Z.H. (2018). Pyridostigmine for the treatment of gastrointestinal symptoms in systemic sclerosis. Semin. Arthritis Rheum..

[68] Robinson P.C., Terkeltaub R., Pillinger M.H., Shah B., Karalis V., Karatza E., Liew D., Imazio M., Cornel J.H., Thompson P.L. (2021). Consensus Statement Regarding the Efficacy and Safety of Long-Term Low-Dose Colchicine in Gout and Cardiovascular Disease. Am. J. Med..

[69] Fisher D.M. (1999). Clinical pharmacology of neuromuscular blocking agents. Am. J. Health Syst. Pharm..

